# EZH2-dependent chromatin looping controls *INK4a *and *INK4b*, but not *ARF*, during human progenitor cell differentiation and cellular senescence

**DOI:** 10.1186/1756-8935-2-16

**Published:** 2009-12-02

**Authors:** Sima Kheradmand Kia, Parham Solaimani Kartalaei, Elnaz Farahbakhshian, Farzin Pourfarzad, Marieke von Lindern, C Peter Verrijzer

**Affiliations:** 1 Department of Biochemistry, Center for Biomedical Genetics, Erasmus University Medical Center, PO Box 1738, 3000 DR Rotterdam, The Netherlands; 2 Department of Hematology, Erasmus University Medical Center, PO Box 1738, 3000 DR Rotterdam, The Netherlands; 3 Department of Cell Biology, Erasmus University Medical Center, PO Box 1738, 3000 DR Rotterdam, The Netherlands

## Abstract

**Background:**

The *INK4b-ARF-INK4a *tumour suppressor locus controls the balance between progenitor cell renewal and cancer. In this study, we investigated how higher-order chromatin structure modulates differential expression of the human *INK4b-ARF-INK4a *locus during progenitor cell differentiation, cellular ageing and senescence of cancer cells.

**Results:**

We found that *INK4b *and *INK4a*, but not *ARF*, are upregulated following the differentiation of haematopoietic progenitor cells, in ageing fibroblasts and in senescing malignant rhabdoid tumour cells. To investigate the underlying molecular mechanism we analysed binding of polycomb group (PcG) repressive complexes (PRCs) and the spatial organization of the *INK4b-ARF-INK4a *locus. In agreement with differential derepression, PcG protein binding across the locus is discontinuous. As we described earlier, PcG repressors bind the INK4a promoter, but not ARF. Here, we identified a second peak of PcG binding that is located ~3 kb upstream of the *INK4b *promoter. During progenitor cell differentiation and ageing, PcG silencer EZH2 attenuates, causing loss of PRC binding and transcriptional activation of *INK4b *and *INK4a*. The expression pattern of the locus is reflected by its organization in space. In the repressed state, the PRC-binding regions are in close proximity, while the intervening chromatin harbouring *ARF *loops out. Down regulation of EZH2 causes release of the ~35 kb repressive chromatin loop and induction of both *INK4a *and *INK4b*, whereas *ARF *expression remains unaltered.

**Conclusion:**

PcG silencers bind and coordinately regulate *INK4b *and *INK4a*, but not *ARF*, during a variety of physiological processes. Developmentally regulated EZH2 levels are one of the factors that can determine the higher order chromatin structure and expression pattern of the *INK4b-ARF-INK4a *locus, coupling human progenitor cell differentiation to proliferation control. Our results revealed a chromatin looping mechanism of long-range control and argue against models involving homogeneous spreading of PcG silencers across the *INK4b-ARF-INK4a *locus.

## Background

**Figure 1:**
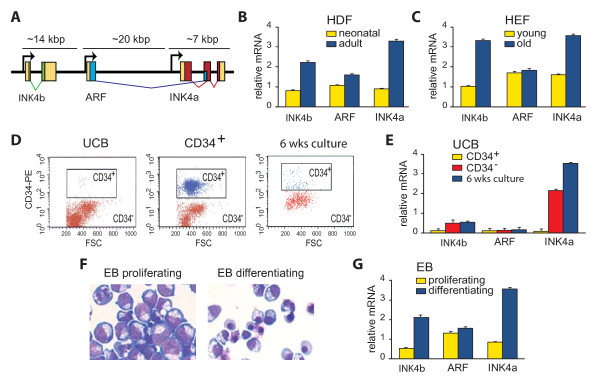
**Expression of the *INK4b-ARF-INKa *locus during cellular ageing and differentiation**. (A) Organization of the human *INK4b-ARF-INK4a *locus (not drawn to scale), encoding three distinct proteins, *p15*^*INK4b*^, *p14*^*ARF *^and *p16*^*INK4a*^. The untranslated regions (yellow boxes), the coding sequences of *p15*^*INK4b *^(green), *p14*^*ARF *^(blue) and *p16*^*INK4a *^(red) are indicated. (B) *INK4b *and *INK4a*, but not *ARF*, are upregulated in ageing human diploid fibroblasts (HDFs). RT-qPCR analysis of *INK4b-ARF-INK4a *expression in neonatal (yellow bar) versus adult (blue bar) HDFs. Bar graphs represent the mean of three independent biological replicate experiments, each analyzed in triplicate by RT-qPCR. mRNA levels are expressed relative to *Gapdh*. Error bars represent standard error of the mean. (C) *INK4b *and *INK4a *are selectively upregulated in ageing human embryonic fibroblasts (HEFs). Comparison of *INK4b-ARF-INK4a *expression in HEF cells (TIG3) at a low passage doubling (PDL 26, yellow) and high PDL (PDL 64, blue). (D) Flow cytometrical analysis of umbilical cord blood cells showing forward scatter (FSC) on the *x*-axes and CD34 staining on the *y*-axes. The immature CD34^+ ^cells (blue) and mature CD34^- ^cells (red) were sorted (left hand panel). Isolated CD34^+ ^cells were reanalysed (middle panel; red dots represent 65% of the population). Following 6 weeks of culture these cells stained negative for CD34 (less than ~15%; right hand panel). (E) *INK4b-ARF-INK4a *expression in CD34^+ ^(yellow) cells, CD34^- ^(red) cells, and the progeny of seeded CD34^+ ^cells after 6 weeks of culture (blue). (F) Erythroblasts (EB) kept under proliferation conditions for 2 days following induction of differentiation towards erythrocytes. Cells were cytocentrifuged onto glass slides and stained for haemoglobin (brown colour) and with standard cytologic dyes. (G) Expression of *INK4b-ARF-INK4a *in proliferating (yellow) erythroblst cells and cells differentiating towards erythrocytes (blue).

Development and homeostasis require the coordinate regulation of cell proliferation and differentiation. The *INK4b-ARF-INK4a *tumor suppressor locus (Figure 1A) plays a central role in controlling the equilibrium between progenitor cell renewal and cancer risk [1-8]. This locus encodes three cell cycle inhibitory proteins: p15^INK4b^, p14^ARF ^and p16^INK4a ^[3,8]. p15^INK4b ^and p16^INK4a ^are closely related proteins and both act on the Rb-pathway through the inhibition of the proliferation-promoting cyclin-dependent kinases CDK4 and CDK6. p14^ARF ^is structurally and functionally unrelated to p15^INK4b^ or p16^INK4a ^and works primarily through activation of the p53 pathway. A large number of studies have suggested a role for the *INK4b-ARF-INK4a *locus in cancer suppression and promotion of ageing. p16^INK4a^, in particular, has been implicated in balancing the need for tissue renewal and the risk of tumourigenesis [1-8]. Perhaps not surprisingly, the regulation of the *INK4b-ARF-INK4a *locus is highly complex. *INK4b-ARF-INK4a *expression is controlled by various signal transduction pathways and patterns of expression vary depending on physiological circumstances. Coordinated regulation of the whole locus, as well as differential gene expression, has been described [3]. Unfortunately, regulation of *INK4b *has received significantly less attention than that of *ARF *and *INK4a*.

The polycomb group (PcG) silencers form an important class of transcriptional corepressors that control the expression of the *INK4b-ARF-INK4a *locus [9]. This was first suggested by the finding that the PcG protein, BMI1, promotes oncogenesis in mice through the silencing of *INK4a *[10]. Since then, other PcG proteins have also been implicated in the silencing of the *INK4b-ARF-INK4a *locus, including the histone H3 lysine 27 (H3K27) methyltransferase EZH2 [9-22]. However, in contrast to *INK4a *and *ARF*, the role of PcG proteins in *INK4b *expression control has not been extensively studied.

PcG proteins function as part of larger multi-protein assemblages, referred to as polycomb repressive complexes (PRCs) [23-25]. One major class is formed by PRC1-like complexes, which include assemblages harbouring BMI1. PRC1 class complexes have been implicated in chromatin compaction and histone H2A ubiquitylation. The second major class is formed by PRC2-like complexes, harbouring histone H3K27 methylases such as EZH2. However, it is important to stress that there is likely to be a great variety of PRCs and additional enzymatic activities. For example, PRC1 subunits are also part of alternate assemblages such as *Drosophila *dRAF [26] and its mammalian relatives [27,28]. The dRAF complex, harbouring the BMI1 homolog PSC, dRING and the demethylase dKDM2, ubiquitylates histone H2A and demethylates histone H3K36 during gene silencing [26]. Very recently, an essential role for N-acetylglucosamination in PcG repression was established [29]. Importantly, PRCs have also been implicated in the higher-order chromatin organization through looping [30,31].

Although the developmental roles of many PcG proteins await further analysis, recent research has emphasized their importance in dynamic gene control during the differentiation of precursor cells, in cancer and cellular senescence [9-22,32-36]. In particular, several of these studies have shown the importance of EZH2 for the dynamic regulation of gene silencing, orchestrating the differentiation of progenitor cells.

Here, we have addressed the role of PcG silencers in the regulation of the human *INK4b-ARF-INK4a *locus during the differentiation of progenitor cells, cellular ageing and cellular senescence of cancer cells. During these diverse physiological processes *INK4b *and *INK4a *were coordinately induced, whereas *ARF *remained unaltered. In order to investigate the underlying mechanisms, we analysed the spatial organization of the *INK4b-ARF-INK4a *locus. Our results revealed long-range control through chromatin looping rather than 'blanket' spreading of PcG proteins across the whole *INK4b-ARF-INK4a *locus. We conclude that PcG proteins control the higher-order chromatin conformation of the *INK4b-ARF-INK4a *locus, providing a molecular mechanism for coupling cell differentiation to proliferation control.

## Results

### Selective induction of INK4b and INK4a during cellular ageing, differentiation and senescence

We compared the expression of the human *INK4b-ARF-INK4a *locus in neonatal and adult human diploid fibroblasts (HDFs). The proliferation rate of adult HDFs is ~2-3 times slower than that of neonatal HDFs. In order to monitor *INK4b-ARF-INK4a *expression, we extracted RNA, which was followed by reverse transcription and real-time quantitative polymerase chain reaction (RT-qPCR) with gene-selective primers (Figure 1B). Compared to neonatal cells (yellow), the expression of *INK4a *and *INK4b*, but not of *ARF*, in adult HDFs (blue) is significantly higher. We also compared human embryonic fibroblasts (HEFs) with a low passage number (PDL 26) with old cells (PDL 64). We observed a gradual, up to two- to threefold, increase in doubling time as the passage number became higher. *INK4a *and *INK4b *are clearly upregulated in HEFs with a high passage number, whereas *ARF *expression remained unchanged (Figure 1C). We conclude that, in these untransformed human diploid cells, *INK4a *and *INK4b*, but not ARF, are coordinately upregulated during ageing.

Next, we examined the expression of the *INK4b-ARF-INK4a *locus in human cells with a broad versus restricted potential for differentiation. We sorted CD34^+ ^and CD34^- ^cells isolated from human umbilical cord blood (UCB; Figure 1D). CD34^+ ^UCB cells comprise quiescent stem cells, but they mainly represent the transiently amplifying compartment of multipotent and early myeloid progenitors [37]. In contrast, the CD34^- ^fraction contains committed cells in late stages of differentiation. A fluorescence activated cell sorting analysis of the isolated CD34^+ ^cells revealed a purity of about 65% (Figure 1D, middle panel). We cultured the purified CD34^+ ^cells for 6 weeks, after which the majority of cells (~85%) matured to CD34^- ^cells, representing mainly postreplicative erythroblasts and granulocytes. An expression analysis of CD34^+ ^(yellow) and CD34^- ^cells (red) immediately following isolation, and CD34^+ ^derived cells cultured for 6 weeks (blue), demonstrated up-regulation of *INK4b *and very strong *INK4a *induction during differentiation (Figure 1E). In contrast, *ARF *expression remained unaltered.

As an alternative way to study the effects of cell differentiation, we cultured erythroblasts (EB) from human fetal liver. After 7 days, EBs were either kept under proliferating conditions or induced to differentiation towards erythrocytes for 2 days (Figure 1F). Again, the differentiation was accompanied by selective activation of *INK4a *and *INK4b *(Figure 1G).

As a model for senescence in cancer cells, we used MON human malignant rhabdoid tumour (MRT) cells. MRTs are caused by the loss of the hSNF5 subunit of the SWI/SNF chromatin remodelling complex [38]. Re-expression of hSNF5 in MRT cells restores SWI/SNF recruitment to *INK4b *and *INK4a*, causing the eviction of PRCs and a loss of silencing [20]. Consequently, these cells first undergo a G1/S cell cycle arrest and later become senescent [20,39]. hSNF5-induced cellular senescence of MRT cells is p16^INK4a^-dependent [20,39]. We suspect that the hSNF5-mediated senescence of MRT cells might be due to the inability of oncogenic stress signalling to activate *INK4a *expression in the absence of hSNF5. In summary, we identified a number of diverse physiological processes in which human *INK4a *and *INK4b *are coordinately induced, while ARF expression remained unaltered.

### EZH2 is down-regulated during progenitor cell differentiation

**Figure 2:**
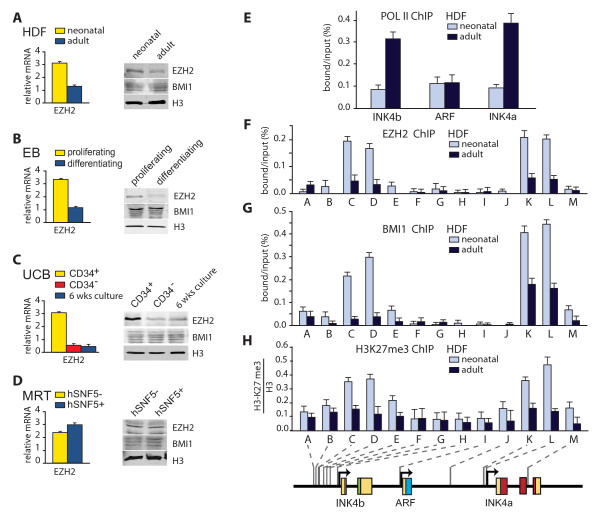
**EZH2 attenuation during progenitor cell differentiation**. (A-D) PRC2 subunit EZH2, but not the PRC1 subunit BMI1, is down-regulated during cellular ageing and differentiation. The expression of EZH2 was analysed by RT-qPCR and Western immunoblotting in: (A) neonatal and adult human diploid fibroblasts (HDFs); (B) proliferating and differentiating erythroblasts, (C) immature CD34^+ ^progenitors and mature CD34^- ^cells isolated directly from umbilical cord blood and CD34^+ ^cells that differentiated and lost CD34 expression following 6 weeks of culture; (D) malignant rhabdoid tumour cells that either lack or express hSNF5. For characterization of these cells see Figure 1. In parallel, BMI1 and histone H3 levels were determined. (E) Selective RNA POL II recruitment to *INK4a *and *INK4b*, but not *ARF*, in ageing HDFs. Chromatin immunoprecipitation (ChIP)-quantitative polymerase chain reaction (qPCR) analysis of RNA POL II binding to the *INK4b-ARF-INK4a *locus in neonatal (light blue) and adult (dark blue) HDFs. The following primer sets were used: H (*INK4b*), I (*ARF*) and L (*INK4a*). All ChIP data presented in this study are the result of at least three biological replicates. Background levels were determined using antibodies directed against GST. The abundance of specific DNA sequences in the immunoprecipitates was analysed by qPCR and corrected for the independently determined amplification curves for each primer set. ChIP signal levels for each region are presented as percentage of input chromatin. Bar graphs represent the mean of three independent experiments, each analysed in triplicate by qPCR. Error bars represent standard error of the mean.(F) PRCs bind *INK4a *and *INK4b *in neonatal but not in adult HDFs. ChIP-qPCR analysis revealed highly localized binding of EZH2 to the *INK4a *promoter region (primer sets K and L) and an area ~3 kb upstream of the *INK4b *promoter (primer sets C and D) in neonatal HDFs. In adult HDFs, EZH2 protein binding is strongly reduced. Analysis was as described above. (G) Following waning of EZH2, BMI1 binding to *INK4a *and *INK4b *is reduced significantly. ChIP-qPCR analysis of BMI1 binding to the *INK4b-ARF-INK4a *locus in neonatal and adult HDFs. The positions of the amplified regions (A-M) of the *INK4b-ARF-INK4a *locus are indicated at the bottom. (H) ChIPs using antibodies directed against histone H3K37 me3 revealed increased H3K27 methylation at- and around the PRC binding sequences upstream of *INK4b *and at the *INK4a *promoter. H3K27 me3 ChIPs were normalized against histone H3.

Because the PcG silencers EZH2 and BMI1 play important roles in the repression of the *INK4b-ARF-INK4a *locus, we investigated their expression in young versus adult HDFs and during cellular differentiation. RT-qPCR and Western immunoblotting revealed lower EZH2 levels in adult HDFs compared to the neonatal cells. In contrast, the BMI1 levels were comparable (Figure 2A). When we compared proliferating EBs with differentiating cells we again observed a reduction of EZH2 levels, but not of BMI1 (Figure 2B). Likewise, UCB CD34^+ ^progenitor cells expressed much higher levels of EZH2 than mature CD34^- ^cells (Figure 2C). Upon culture, CD34^+ ^cells matured and, concomitantly, EZH2 expression was strongly attenuated, whereas BMI1 levels remained stable (Figure 2C). However, re-expression of hSNF5 in MRT cells mediates *INK4a *and *INK4b *induction without affecting EZH2 or BMI1 levels [20]. We conclude that, in differentiating haematopoietic progenitor cells, the expression of the PRC2 subunit EZH2 wanes. In contrast, levels of the PRC1 subunit BMI1 remained constant.

In order to determine the effects of EZH2 down-regulation on chromatin occupancy at the *INK4b-ARF-INK4a *locus, we used chromatin immunoprecipitations (ChIPs) monitored by qPCR. Comparing neonatal and adult HDFs revealed an increased recruitment of RNA polymerase II (RNA POLII) to *INK4a *and *INK4b *in adult cells, consistent with their enhanced transcription (Figure 2E). Next, we established the pattern of EZH2 and BMI1 binding, and the relative level of histone H3K27 me3 (Figure 2F-H). PRC binding at the *INK4a *promoter is already well established. We performed a detailed analysis of EZH2 and BMI1 binding to the *INK4b *upstream region and used a selection of primers targeting *INK4a *and *ARF *we published earlier [20] to serve as a reference. In addition to the *INK4a *promoter region (primer sets K and L), we identified a second peak of PRC binding to an area ~3 kb upstream of the *INK4b *promoter (primer sets C and D). Outside these two domains, binding of EZH2 and BMI1 across the *INK4b-ARF-INK4a *locus was low. H3K27 me3 levels follow EZH2 binding but are spread over a larger area (Figure 2H). Although only EZH2 was down-regulated in adult HDFs, both EZH2 and BMI1 occupancy at *INK4a *and *INK4b *was strongly reduced in these cells (Figure 2F and 2G). This observation agrees well with earlier studies showing that EZH2 can facilitate binding of other PcG proteins [23-25,40]. Like EZH2 binding, H3K27 me3 levels were strongly reduced in adult HDFs compared to neonatal cells (Figure 2H). In senescing MRT cells a distinct mechanism operates. hSNF5 re-expression does not affect EZH2 expression (Figure 2D), but enables SWI/SNF recruitment to *INK4a *and *INK4b*, leading to PRC eviction [20].

### EZH2 is required for coordinate silencing of INK4a and INK4b

**Figure 3:**
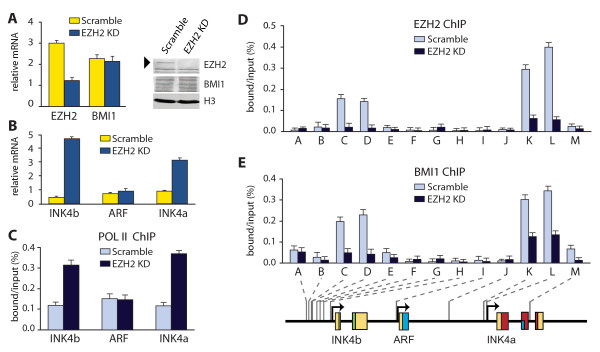
**Loss of EZH2 causes derepression of *INK4a *and *INK4b***. (A) Neonatal human diploid fibroblasts were transduced with lentiviruses expressing either shRNAs targeting *E(z)h2 *mRNA (EZH2 KD) or a scrambled control. Three days following transduction, EZH2 levels were analysed by real-time quantitative polymerase chain reaction (RT-qPCR) and Western immunoblotting, as described above. The band corresponding to EZH2 is indicated with an arrowhead. In parallel, BMI1 and histone H3 levels were determined. (B) Loss of EZH2 causes transcriptional activation of *INK4b *and *INK4a*. Seventy-two hours following transduction, relative expression levels of *INK4b*, *ARF *and *INK4a *were determined by RT-qPCR of isolated mRNA. (C) EZH2 depletion leads to RNA POL II recruitment to *INK4a *and *INK4b*, as determined by chromatin immunoprecipitation (ChIP)-qPCR. (D-E) EZH2 depletion causes loss of PRCs from *INK4a *and *INK4b*, as revealed by ChIP-qPCR using antibodies directed against EZH2 (D) or BMI1 (E). All analyses were as described in the legend to Figures 1 and 2.

Our results showed that attenuation of EZH2 is accompanied by a loss of PRC1 and PRC2-binding, recruitment of RNA POL II and induction of *INK4a *and *INK4b*. *ARF *expression remained unaffected and is not co-regulated with *INK4a *and *INK4b *in the human cells studied here. These observations suggest a critical role for EZH2 in the selective regulation of *INK4a *and *INK4b *expression, during ageing and differentiation. To test whether EZH2 is indeed required for the silencing of *INK4a *and *INK4b*, we used a shRNA strategy to attenuate its levels in neonatal HDFs. Cells were transduced with lentiviruses expressing either shRNAs targeting EZH2 mRNA (EZH2 KD) or a scrambled control. Three days following the transduction, EZH2 levels were effectively reduced in cells treated with the appropriate shRNA (Figure 3A). In contrast, BMI1 levels were unaffected. Loss of EZH2 caused a strong induction of *INK4a *and *INK4b *but not of *ARF *(Figure 3B). RNA POL II was selectively recruited to the *INK4a *and *INK4b *loci, as revealed by ChIP-qPCR (Figure 3C). Depletion of EZH2 leads to its expected disappearance from *INK4a *and *INK4b*, but also causes loss of BMI1 binding (Figure 3D and 3E). Similar results were obtained in MRT cells (Additional file 1, Figure S2). Taken together, these results suggest that EZH2 attenuation is sufficient for the dissociation of PcG silencers and induction of *INK4a *and *INK4b*.

### A chromatin loop, linking the repressed INK4a and INK4b, is released during induced expression

**Figure 4:**
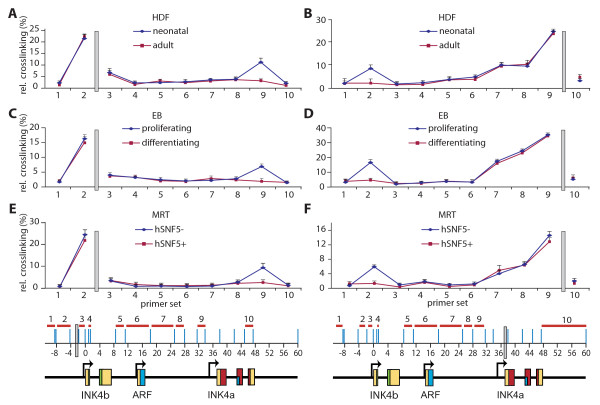
**Regulated chromatin looping between *INK4a *and *INK4b***. (A-F) Chromatin conformation capture - quantitative polymerase chain reaction (qPCR) analyses of long-range interactions at the *INK4b-ARF-INK4a *locus. Locus-wide relative cross-linking frequencies of EcoRI fragments 1 to 10 are plotted. The constant primer and the TaqMan probe (grey bar) were either in the EcoRI fragment -4 to -2 kb upstream of *INK4b *(A, C and E) or overlapping the *INK4a *promoter (B, D and F). The locations within the *INK4b-ARF-INK4a *genomic region of the EcoRI sites (blue lines), bait fragment (harbouring the grey bar, indicating the TaqMan probe) and suitable candidate EcoRI fragments (numbered red segments) are shown at the bottom. The relative cross-linking frequency of each fragment is plotted, with the fragment numbers indicated on the X-axis. (A, B) Neonatal (blue) and adult (red) human diploid fibroblasts. (C, D) Proliferating- (blue) and differentiating (red) eryhtroblasts. (E, F) Proliferating MRT cells lacking hSNF5 (blue) and senescing MON cells expressing hSNF5 (red). Each point represents the mean of three independent biological replicates, each analysed in triplicate by qPCR. Standard deviations are depicted by error bars.

We wondered whether, because of their coordinate regulation by PcG silencers, *INK4a *and *INK4b *might be in close physical proximity, in spite of their being over 35 kb apart. In order to investigate the three-dimensional conformation of the *INK4b-ARF-INK4a *locus, we used chromatin conformation capture (3C) technology in combination with qPCR [41,42]. We first compared the *INK4b-ARF-INK4a *locus higher-order chromatin structure in neonatal and adult HDFs (Figure 4A). Cells were cross-linked with formaldehyde, followed by chromatin isolation and restriction digestion with EcoRI. Our preliminary analysis yielded 10 suitable EcoRI fragments across almost 70 kb of DNA encompassing the *INK4b-ARF-INK4a *locus (see Methods). Samples were ligated under conditions that favour the union of DNA fragments that are physically connected and qPCR across junctions was used to determine the relative cross-linking frequency between restriction fragments. All 3C data presented here are the result of three independent biological replicate experiments. The 'constant' primer and the TaqMan probe (grey bar) were designed in the EcoRI fragment ~4 kb to 2 kb upstream of *INK4b*, harbouring the PRC-binding sequences. Plotting of the ligation frequencies to this 'bait' fragment revealed a clear peak at fragment 9 overlapping the *INK4a *promoter proximal region. These experiments were complimented by 3C analysis using a bait fragment near the *INK4a *promoter. Now, we observed a peak at fragment 2, encompassing the PRC-binding domain upstream of the *INK4b *promoter (Figure 4B). We conclude that, in neonatal HDFs, the repressed *INK4b-ARF-INK4a *locus has a looped structure. The PRC-bound regions upstream of *INK4b *and proximal to the *INK4a *promoter are close in nuclear space, whereas the ~35 kb of intervening DNA, including the *ARF *promoter, loops out.

In adult HDFs, the higher-order chromatin conformation of the *INK4b-ARF-INK4a *locus is dramatically different. 3C-qPCR analyses revealed a loss of long-range interaction between *INK4a *and *INK4b*, suggesting the locus adopted a linear conformation (Figure 4A-B). In order to study the effects of progenitor cell differentiation, we undertook a similar 3C analysis of the chromatin structure in proliferating and differentiating ERs (Figure 4C and 4D). Our results revealed the presence of a chromatin loop between *INK4a *and *INK4b *in proliferating cells, which is similar to that in neonatal HDFs. When ERs were induced to differentiate towards erythrocytes, the silent chromatin loop dissolved and *INK4a *and *INK4b *became de-repressed. Finally, we compared proliferating MRT cells with senescing cells after hSNF5 expression (Figure 4E and 4F). Following hSNF5 expression and PRC removal, the repressive chromatin loop is released and the *INK4b-ARF-INK4a *locus assumes a linear conformation.

Together, our results showed that, in neonatal HDFs, haematopoietic progenitor cells and MRT cancer cells, the repressed *INK4b-ARF-INK4a *locus assumes a looped conformation. The ~35 kb chromatin loop links the PRC-binding regions of *INK4a *and *INK4b*, whilst excluding *ARF*. Concomitant with the increased transcription of *INK4a *and *INK4b *in adult HDFs, following differentiation or senescence, the chromatin loop dissolves. The loss of looping is concomitant with the loss of PRC-binding, which, in HDFs and differentiating EBs, is caused by attenuation of EZH2. In MRT cells driven towards senescence, EZH2 levels remain stable, but the PRCs are removed by SWI/SNF action. This duly leads to a release of the repressive loop and gene activation, again suggesting that PRC-binding is required for looping.

### PRC-binding is required for looping between INK4a and INK4b

**Figure 5:**
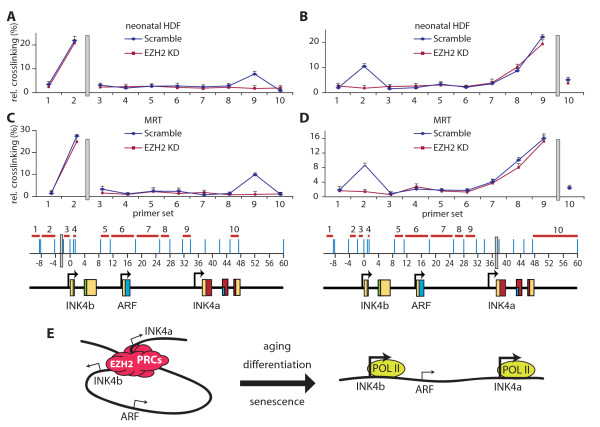
**EZH2 is required for looping between *INK4a *and *INK4b***. (A-D) Chromatin conformation capture-quantitative polymerase chain reaction (PCR) analysis on neonatal human diploid fibroblasts (A, B) and malignant rhabdoid tumour (MRT) cells (C, D) transduced with lentiviruses expressing either shRNAs targeting *E(z)h2 *mRNA (red) or a scrambled control (blue). Procedures were as described in the legend to Figure 4. (E) Parsimonious model for the coordinate regulation of human INK4a and INK4b by PcG repressive complexes (PRCs). In multipotent progenitor cells, young fibroblasts and highly proliferating MRT cancer cells, PRCs simultaneously bind the INK4a promoter and the INK4b upstream promoter region. This creates a looped chromatin structure that links the silenced INK4b and INK4a, but excludes ARF. EZH2 is critical for PRC binding and silencing. During progenitor cell differentiation and ageing, EZH2 attenuates, causing reduced locus occupancy of PRCs and release of the repressive loop. Concomitantly, RNA POL II is recruited selectively to INK4b and INK4a, but not to ARF, leading to gene transcription. MRT cells contain a crippled SWI/SNF complex, due to loss of the hSNF5 tumor suppressor subunit. hSNF5 re-expression in these cells restores SWI/SNF targeting to INK4a and INK4b, causing PRC eviction and release of the repressive loop. For details see discussion.

To test whether EZH2 is crucial for loop formation, we transduced neonatal HDFs with lentiviruses expressing either a shRNA targeting EZH2 mRNA or a scrambled control. As shown above, depletion of EZH2 leads to a loss of PRC binding to *INK4a *and *INK4b *upstream regions (Figure 3). 3C-qPCR analyses revealed that a loss of EZH2 also results in the release of the repressive chromatin loop that links *INK4a *and *INK4b *(Figure 5A and 5B). As shown above, the restoration of SWI/SNF targeting in MRT cells provides an alternate mechanism of PRC removal and loop release (Figure 4E and 4F; Additional file 1, Figure S1). However, EZH2 depletion in the absence of hSNF5 expression, also led to loss of EZH2 and BMI1 binding (Additional file 1, Figure S2) and loss of looping (Figure 5C and 5D). Taken together, these results show that PRC binding is critical for chromatin looping at the *INK4b-ARF-INK4a *locus. When PRC binding is lost, due either to diminished EZH2 levels or because of SWI/SNF action in MRT cells, the repressive chromatin loop is released concomitant with *INK4a *and *INK4b *induction (Figure 5E).

## Discussion

### PcG proteins control the balance between differentiation and proliferation

Our results and those of others [18,34,36,40,43], emphasize a crucial role for EZH2 in orchestrating progenitor cell differentiation and proliferation control. Ezhkova *et al*. [18] observed derepression of *INK4a *and *INK4b *due to EZH2 down-regulation, controlling the balance between the proliferative basal layer of progenitor cells and non-proliferating differentiated epidermal cells. These results in the mouse epidermal lineage are highly reminiscent of our findings in human haematopoietic progenitor cells. The histone H3K36 demethylase JHDM1b/KDM2b, a mammalian homolog of the dRAF signature subunit dKDM2, binds and regulates *INK4b *[19]. Reminiscent of the results described here, He *et al*. [19] observed that a knockdown of JHDM1b/KDM2b or RING1b in primary mouse embryo fibroblasts causes induction of *INK4b *and *INK4a *but not of ARF or the p53-pathway. Consequently, these cells undergo arrest and cellular senescence, for which p15^INK4b^, it turns out, are critical. In response to oncogenic RAS-signalling in human fibroblasts, the H3K27 me3 demethylase JMDJ3/KDM6B activates *INK4a*, but not *ARF *[11,13]. In mouse embryo fibroblasts, JMJD3/KDM6B activates both *INK4a *and *ARF*, possibly reflecting a difference in *INK4/ARF *regulation between mice and man [3,44]. However, as mentioned above, other studies have also presented induction of *INK4a *and *INK4b*, but not *ARF *in mouse cells (see, for example, [18] and [19]). Here, we identified EZH2- and BMI1-binding sequences upstream of the *INK4b *promoter, providing additional evidence for the control of *INK4b *by PcG repression. Finally, we emphasize that, under different physiological conditions, different combinations of *INK4b-ARF-INK4a *expression will be relevant. See, for example, the many examples of co-induction of *ARF *and *INK4a *[3,7,8]. Related to this point, it is easy to imagine that, in those situations, the *INK4b-ARF-INK4a *locus might assume a more distinct conformation than that described here.

Regulation of *INK4a *and *INK4b *by EZH2 might be a conserved mechanism required to balance progenitor cell proliferation and differentiation. Unfortunately, this coupling might have sinister consequences when control of EZH2 expression is lost [40]. EZH2 is over-expressed in a variety of tumours, potentially blocking the tumour suppression function of *INK4b-ARF-INK4a*. In these cells, EZH2 seems to promote de-differentiation and uncontrolled proliferation. Notably, the expression level of EZH2 in early murine haematopoietic cells correlates with their expansion potential. EZH2 overexpression in early haematopoietic progenitors led to a loss of their repopulating ability [43]. Together with early studies in *Drosophila*, these observations emphasize the importance of PcG protein regulation and dosage.

Following the hSNF5 expression in MRT cells, which triggers the cellular senescence programme, EZH2 levels do not change. In these cells, the SWI/SNF chromatin remodeller is crippled due to a loss of hSNF5 and, therefore, unable to evict PRCs from *INK4a *and *INK4b *regulatory elements [20]. Following hSNF5 expression, SWI/SNF is able to remove PRCs from *INK4a *and *INK4b*, causing the release of the repressive chromatin loop and gene expression. Thus, a loss of function of a remodeller that counteracts PcG silencing may have similar consequences as EZH2 over-expression. Together with the earlier identification of SWI/SNF as a suppressor of *PcG *mutations in flies [23,45], these findings emphasize the evolutionary conservation of this regulatory antagonism.

### Higher-order chromatin structure and gene expression control

Several studies have implicated higher-order chromatin structure in the regulation of complex multi-gene loci [30,31,46-51]. Typically, models explaining gene control at a distance invoke either spreading of factors along a chromatin fibre or long-range protein-protein interactions that cause looping-out of the intervening chromatin. Two earlier studies proposed a continuous spreading across the whole *INK4b-ARF-INK4a *locus of either heterochromatinization [52] or PcG proteins [15]. However, the ChIP data in the latter study actually showed a clear peak of PRC binding at the INK4a promoter, which tapers off and is near background at the ARF gene. This pattern of binding at *INK4a *is reminiscent of what we observed [20]. For the cell types and physiological processes studied here, we favour a discontinuous looping mechanism of *INK4b-ARF-INK4a *locus control over models invoking blanket spreading of silencers. First, there are two distinct peaks of PRC binding: first, ~3 kb upstream of the *INK4b *promoter and, second, at the *INK4a *promoter. Outside these domains, PRC binding is near background levels and we detected no significant PRC-binding at *ARF*. We note that the PRC-binding peak upstream of INK4b, albeit that it is nearby, does not coincide with the RD regulatory region identified by Gonzalez *et al*. [52]. In addition, *INK4a *and *INK4b *are coordinately derepressed during cell differentiation, ageing and senescence, whereas *ARF *remained unaffected. Finally, the PRC-bound *INK4a *and *INK4b *regulatory regions are linked in nuclear space. Loss of PRC-binding causes the release of chromatin looping and induction of INK4a and INK4b.

Our findings for the INK4b-ARF-INK4a locus dove-tail well with other studies revealing PcG-mediated chromatin looping [30,31]. Chromatin looping is a consequence of the association of proteins bound to two or more distal regulatory elements (see, for example, [50,51,53-55] and references therein). When two genes are brought into the same microenvironment, such as the PRC bound *INK4a *and *INK4b*, it facilitates coordinated regulation. The looping-out of an intervening gene, such as *ARF*, provides a physical separation which allows independent regulation. Thus, in our view, the looped-out chromatin per se is neither active nor repressive. Rather, transcriptional consequences are determined locally, by repressors or activators that can alter the local chromatin status. Here, we presented an example where the higher-order chromatin conformation of a human multi-gene locus reflects its differential pattern of gene expression during diverse physiological processes.

## Conclusion

Two classic problems in the study of transcription are the mechanism of long-range gene control and the regulation of multi-gene loci. Here, we studied how PcG silencers control expression of the human *INK4b-ARF-INK4a *tumour suppressor locus. We concentrated on a variety of physiological processes during which *INK4a *and *INK4b *are coordinately upregulated, whereas *ARF *expression remains unaltered. In agreement with differential regulation, our ChIP analysis revealed non-homogeneous PRC binding to the *INK4b-ARF-INK4a *locus. In addition to the *INK4a *promoter, we identified a PRC-binding sequence ~3 kb upstream of the *INK4b *promoter. PRC binding to these two regions mediates the formation of a ~35 kb chromatin loop, linking *INK4b *and *INK4a *but excluding *ARF*. EZH2 attenuation causes the release of the repressive loop and upregulation of *INK4a *and *INK4b*. Thus, EZH2 levels determine the higher-order chromatin structure and expression pattern of the *INK4b-ARF-INK4a *locus, coupling human progenitor cell differentiation to proliferation control. Our findings revealed a looping mechanism of *INK4b *and *INK4a *control, but are difficult to reconcile with models invoking the continuous spreading of PcG silencers.

## Methods

### Cell isolation, cell culture and lentiviral procedures

UCB was collected in 10 ml Hanks+Hepes (H+H) with 1% heparin by nursing staff of the Department of Obstetrics and Gynecology at the Sint Franciscus Hospital, Rotterdam, The Netherlands, following informed consent of the mothers. Mononuclear cells were isolated by Ficoll density centrifugation (Lymphoprep, Nycomed Pharma, Oslo, Norway). The cell suspension was washed twice with Hanks Balanced Salt Solution (HBSS, Gibco, Breda, The Netherlands) and CD34^+ ^cell were isolated using the indirect CD34^+^Microbead kit (Miltenyi Biotec, Germany). The purity of these CD34^+ ^cells was 65-70% as determined by flow cytometry. CD34^+ ^cells were cultured at a density of 1-3 × 10^4^/ml in serum free enriched DMEM as previously described [56]. Medium was supplemented with 100 ng/ml stem cell factor (SCF), 100 ng/ml Flt3-L, and 20 ng/ml trombopoietin. Cells were cultured for 6 weeks and the medium was refreshed every week. In order to establish EB cultures, human fetal liver tissue was obtained from elective abortions of patients who had previously signed informed consent forms giving permission for them to be used for research studies (protocol approved by Erasmus MC medical ethical committee). Fetal livers of 12-18 week human embryos were dissected and passed through a 70-μm nylon mesh. EBs were cultivated at a density of 1-2 × 10^6 ^cells/ml in serum-free medium (StemSpan; Stem Cell Technologies, BC, Canada) enriched with lipids lipids (40 ng/ml cholesterol-rich lipid mix; Sigma) and supplemented with erythropoietin (2 U/ml, a gift of Orthobiotech, Tilburg, The Netherlands), dexamethasone (1 μM; Sigma, MO, USA) SCF (50 ng/ml, supernatant of CHO producer cells) [57]. The EB culture was expanded by daily partial medium changes and the addition of fresh factors, keeping cell density between 1.5-2 × 10^6 ^cells/ml. Proliferation kinetics and size distribution of the cell populations were monitored daily using an electronic cell counter (CASY-1, Schärfe-System, Reutlingen, Germany). In order to induce terminal differentiation EBs were washed and reseeded at 1.5-2 × 10^6 ^cells/ml in lipid-enriched StemSpan supplemented with Epo (5 U/ml) and iron-loaded transferrin (1 mg/ml; SCIPAC Ltd, Kent, UK) [57]. Differentiating EBs were maintained at 2-3 × 10^6 ^cells/ml and harvested 48 h after induction. Cell morphology was analysed in cytospins stained with histological dyes and neutral benzidine [58], using an OlympusBx40 microscope (40× objective, NA 0.65), an OlympusDp50 CCD camera and Viewfinder Lite 1.0 acquisition software. Tissue culture of HDFs and MON MRT cells was performed according to standard protocols. Adult HDFs (Cascade Biologics, Oregon, USA, CAT No. C-004-5C), were isolated from adult human fore skin and have the potential for ~12 population doublings. Neonatal HDFs (Cascade Biologics, CAT No. C-004-5C), were isolated from neonatal foreskin and have the potential for ~16 population doublings. For our experiments, we used adult and neonatal HDFs of comparable, early passages. TIG3 cells were obtained from the Health Science Research Resource Bank (Osaka, Japan, http://cellbank.nibio.go.jp/celldata/jcrb0506.htm). HDF, MON and TIG3 cells were grown in DMEM supplemented with 10% (v/v) FCS. The hSNF5 expressing lentiviral vector has been described [39]. High titre vector stocks were produced in 293T cells by co-transfection of transfer vector constructs with packaging constructs using standard transfection procedures [59]. To deplete EZH2, cells were transduced with lentiviruses expressing shRNA directed against *EZH2 *(Clone TRCN0000040073 and TRCN0000040075; Expression Arrest™-The RNAi consortium (TRC) Human shRNA library purchased from Open Biosystems, USA) for 3 days. In a control experiment, the cells were transduced with scramble, non-targeting lentiviruses.

### Cell extracts and western blotting

Cell extracts were prepared in RIPA buffer (10 mM Tris-HCl, pH 7.5, 150 mM NaCl, 1% Nonidet P-40, 1% NaDOC, 0.1% SDS) and protein concentration determined. ~50 μg of extract was resolved SDS-PAGE, and transferred to 0.45 μm nitrocellulose membrane. Haematopoetic progenitor cells were lysed directly in an equal volume of 2× SDS loading buffer (1 × Tris.Cl pH6.8, 20% glycerol, 4% SDS, O.2 M DTT, 0.001% bromophenol blue). After transfer, membranes were blocked for 1 h in T-PBS with 5% milk and 0.1% Tween20 prior to incubation with primary (overnight) and secondary (1 h) antibodies. Primary antibodies: SUZ12 (Abcam, ab12073), BMI1 (Abcam; ab14389), EZH2 (Santa Cruz; Sc-25383) and Histone H3 (Abcam; ab1791). Western blots were developed with the ECL detection kit (PIERCE) or visualized with the IRDye 680/800 CW (LI-COR) and ODYSSEY Infrared Imageing System according to the supplier's instructions.

### RT-qPCR and ChIP-qPCR assays

Total RNA was extracted from cells using the SV Total RNA Isolation System (Promega, WI, USA). cDNA was synthesized from 1 μg of total RNA using random hexamers and Superscript™ II RNase H-Reverse Transcriptase (Invitrogen). Quantitative real-time PCR (MyIQ, Bio Rad) was performed using SYBR Green I. PCR primers were designed using Beacon designer (Premier Biosoft). The qPCR Core Kit (Invitrogen) was used with 400 nM of each primer under the following cycling conditions: 3 min. at 95°C followed by 40 cycles of 10 s at 95°C and 45 s at 60°C. *Gapdh *was used as an endogenous reference for normalization. Enrichment of specific sequences was calculated using the comparative C_T _method [60]. ChIPs were performed essentially as described by the Upstate protocol http://www.upstate.com. Cross-linked chromatin was prepared from ~2 × 10^7 ^cells. Cells were treated with 1% formaldehyde for 20 min at room temperature. Chromatin isolation, sonication yielding fragments of 300-600 bp and immunoprecipitations were performed according to protocol. The following antibodies were used: BMI1 (Abcam; ab14389), EZH2 (Santa Cruz; Sc-25383), POL II (Santa Cruz; Sc-899), Histone H3 (Abcam; ab1791) and H3-K27 me3 (Upstate; 07-449). The abundance of specific DNA sequences in the immunoprecipitates was determined by qPCR and corrected for the independently determined amplification curves of each primer set. ChIPs using species and isotype-matched immunoglobins directed against an unrelated protein (GST) were used to determine background levels analysed by qPCR as described above. Enrichment of specific DNA sequences was calculated using the comparative C_T _method [60]. ChIP levels for each region are presented as percentage of input chromatin. H3K27 me3 ChIPs were normalized against histone H3. All data presented are the result of at least three biological replicate experiments. PCR primer sequences are provided in Additional file 1, Table S1. Statistical analysis was performed using R software http://www.r-project.org/.

### Chromatin conformation capture assay

The 3C-qPCR assay was performed as described [61]. Formaldehyde-fixed nuclei, prepared from ~10^7 ^cells, were digested with EcoRI overnight, followed by ligation with T4 DNA ligase at 16°C for 4 h. Cross-links were reversed and DNA was purified. The PCR control template, primer efficiency and ligation efficiency were determined by digesting and ligating a BAC clone, which encompassed the entire *INK4b-ARF-INK4a *locus, as previously described [61]. To correct for differences in the quality and quantity of templates, ligation frequencies between the fragments were normalized to a fragment in the human *Ercc3 *locus. Sample purity and digestion efficiency has been carefully assessed as described [61]. Quantification of ligated products was performed by real-time qPCR with a Taqman probe corresponding to a sequence within a DNA fragment overlapping the PRC-binding region of either *INK4b *or *INK4a*. Our analysis yielded 10 suitable EcoR1 fragments covering the *INK4b-ARF-INK4a *locus. The primers and probe sequences are listed in Additional file 1, Table S2. The amplification conditions used in 3C assays are available on request. Cross-linking frequencies were calculated as previously described [51,61].

## Abbreviations

ChIP: chromatin immunoprecipitation; EB: erythroblast; HDF: human diploid fibroblast; HEF: human embryonic fibroblasts; MRT: malignant rhabdoid tumour; PcG: polycomb group; PCR: polymerase chain reaction; PRC: PcG repressive complex; RT-qPCR: real-time quantitative PCR; SCF: stem cell factor; UCB: umbilical cord blood; 3C: chromatin conformation capture; H3K27: H3 lysine 27.

## Competing interests

The authors declare that they have no competing interests.

## Authors' contributions

SKK and CPV conceived and designed the study and wrote the manuscript. SKK and PSK performed most of the experiments. EF, FP and MvL participated in the experiments involving haematopoietic cells and the writing of the parts describing them. All authors have read and approved the manuscript

## Supplementary Material

Additional file 1 Supplementary tables and figures. Click here for file
