# Supplementary material for: EZH2-dependent chromatin looping controls INK4a and INK4b, but not ARF, during human progenitor cell differentiation and cellular senescence
Source: Epigenetics Chromatin. 2009 Dec 2;2:16. doi: 10.1186/1756-8935-2-16 (PMC3225837; doi:10.1186/1756-8935-2-16)
Supplement: Additional file 1 — Supplementary tables and figures. [file 1756-8935-2-16-S1.PDF]

LEGENDS TO SUPPLEMENTARY FIGURES

Supplementary Figure 1. Re-expression of hSNF5 leads to removal of PRCs from *INK4a* and *INK4b*

ChIP-qPCR analysis revealed highly localized binding of BMI1 (A) and EZH2 (B) to the *INK4a* promoter region (primer sets L and M) and an area ~3 kb upstream of the *INK4b* promoter (primer sets C and D) in MON MRT cells. Restoration of SWI/SNF function following hSNF5 expression causes strongly reduced BMI1 and EZH2 binding. Analysis was as described in the legend to Figure 2.

Supplementary Figure 2. Loss of EZH2 causes derepression of *INK4a* and *INK4b* in MRT cells

EZH2 depletion in MON MRT cells due to transduction with lentiviruses expressing either shRNAs targeting *E(z)h2* mRNA (EZH2 KD) or a scrambled control causes loss of EZH2 (A) and BMI1 (B) from *INK4a* and *INK4b*, as revealed by ChIP-qPCR. Analysis was as described in the legends to Figures 2 and 3.

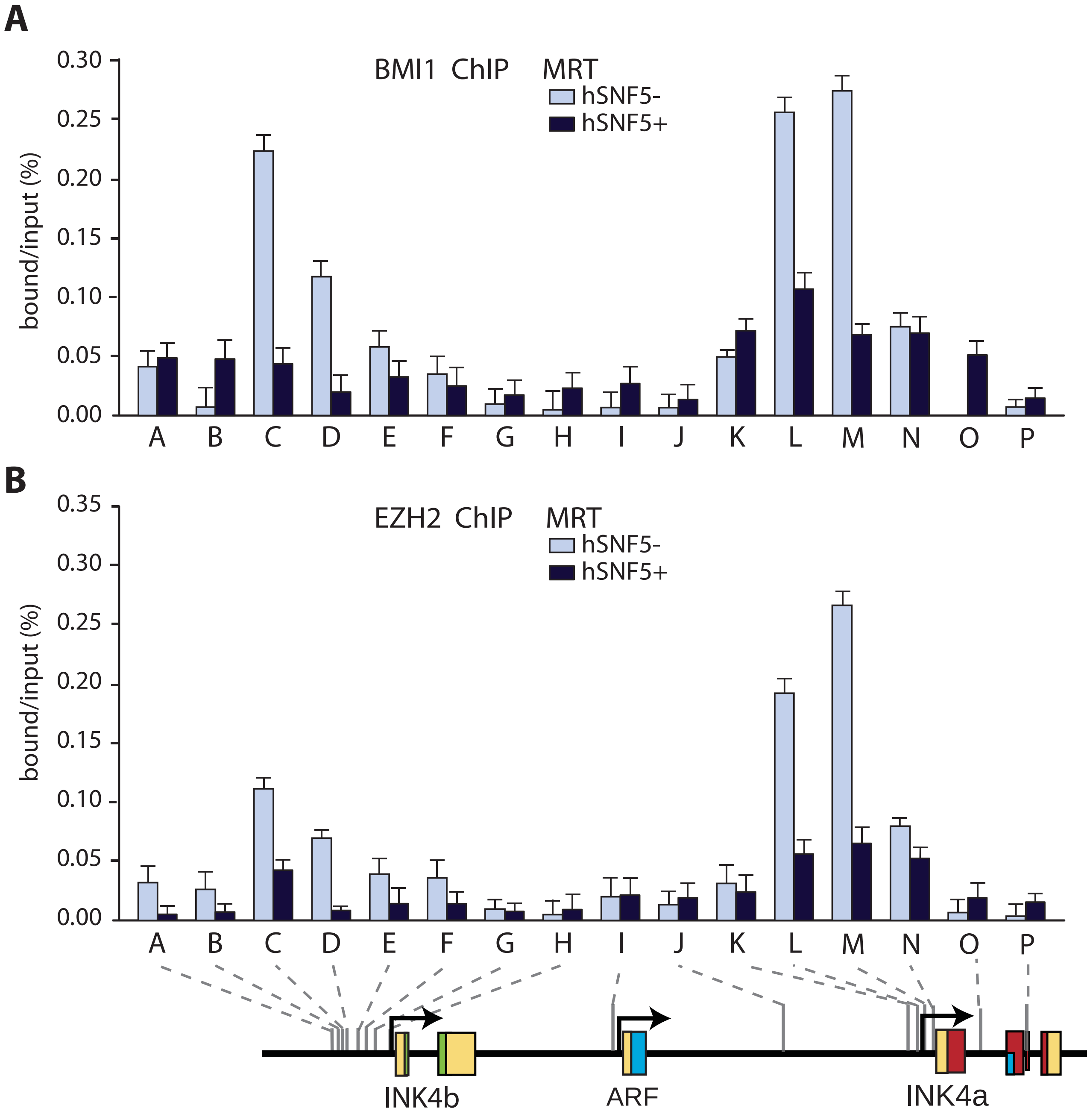
A
BMI1 ChIP MRT
hSNF5-
hSNF5+
bound/input (%)
0.30
0.25
0.20
0.15
0.10
0.05
0.00
A B C D E F G H I J K L M N O P
B
EZH2 ChIP MRT
hSNF5-
hSNF5+
bound/input (%)
0.35
0.30
0.25
0.20
0.15
0.10
0.05
0.00
A B C D E F G H I J K L M N O P
INK4b
ARF
INK4a

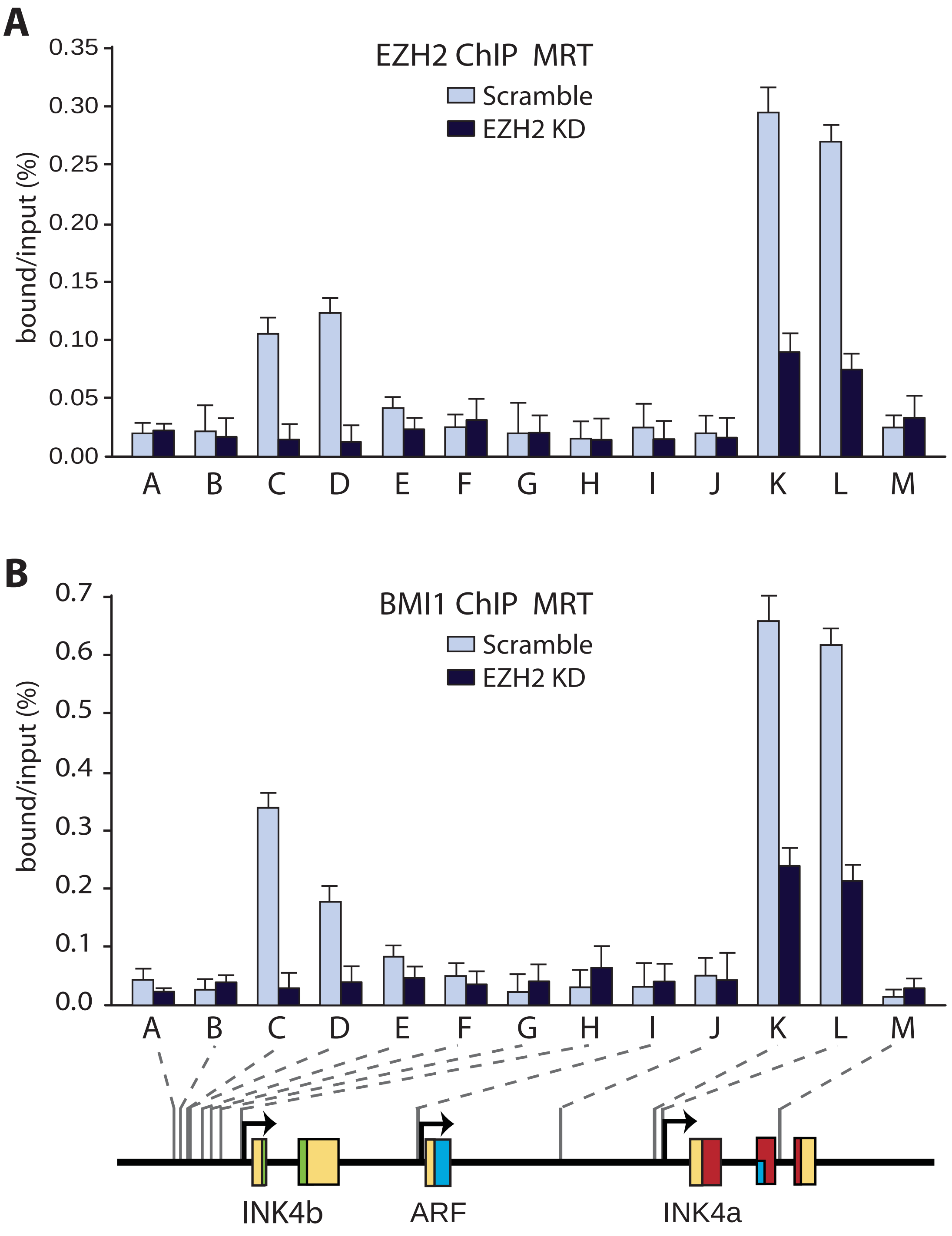
A
EZH2 ChIP MRT
Scramble
EZH2 KD
bound/input (%)
0.35
0.30
0.25
0.20
0.15
0.10
0.05
0.00
A B C D E F G H I J K L M
B
BMI1 ChIP MRT
Scramble
EZH2 KD
bound/input (%)
0.7
0.6
0.5
0.4
0.3
0.2
0.1
0.0
A B C D E F G H I J K L M
INK4b
ARF
INK4a

Table1. Primers used for (A) ChIP, (B) mRNA expression, along the INK4-ARF locus

A: Primers used for ChIP (q PCR) along the INK4-ARF locus

Primer set	Location	Sequence
A	P15 ^{INK4b} (-3,982)	5′- AGTCCTAAGCCCAATACCTCAC
		5′- CTGCCTCTTCACATAGTCATCC
B	P15 ^{INK4b} (-3,575)	5′- ATCACGGAGCAATAAACCCAAC
		5′- CAAGAGAAACAGCGACCTAACC
C	P15 ^{INK4b} (-3,072)	5′- GGGTGGGCTGTTTCTGGAC
		5′- CCTCACGGGCAAGACCATC
D	P15 ^{INK4b} (-2,860)	5′- CCCACTATGTTCCCATCCACTTC
		5′- CCTCACGGGCAAGACCATC
E	P15 ^{INK4b} (-1,476)	5′- GACACATGCCACAGAGGAG
		5′- AGAAGACGAGAAGAGAAATCAAAC
F	P15 ^{INK4b} (-1,065)	5′- AGAAACTGAAGACTAGGAAATGGG
		5′- CTGGACAGGGAAGGGAACC
G	P15 ^{INK4b} (-1,065)	5′- ACTTGCGGTTCTCTTCCTATCC
		5′- TGTGGTGCTGGGCTTGTC
H	P15 ^{INK4b}-Pm	5′-GGCAGTGGTGAACATTCC
		5′-GCCCAAAGATGCTAGGAC
I	P14 ^{ARF}-Pm	5′-CGCCGTGTCCAGATGTCG
		5′-TGCTCTATCCGCCAATCAGG
J	P16 ^{INK4a} (-8.7 kb)	5′-ACTAGGCTTGTCCCACTTGC
		5′-TCAGTTCCTCTCTCCATTCTCC
K	P16 ^{INK4a} (-0.3 Kb)	5′-GGGCTCTCACAACTAGGAAAG
		5′-GGGTGTTTGGTGTCATAGGG
L	P16 ^{INK4a} (+85 bp)	5′-CCCCTTGCCTGGAAAGATAC
		5′-AGCCCCTCCTCTTTCTTCCT
M	P16 ^{INK4a} (+5.6 Kb)	5′- ACCAAGACTTCGCTGACC
		5′-CAAGGAGGACCATAATTCTACC

B: Primers used for mRNA expression (q PCR) of the INK4-ARF locus

P15 ^{INK4b}	5′- ATCACATGAGGTCAGGAGTTCG
	5′- CCAGGTTCAAGCGAGTCTCC
P14 ^{ARF}	5′- GGTTTTCGTGGTTCACATCC
	5′- CCTAGACGCTGGCTCCTC
P16 ^{INK4a}	5′-CCCCTTGCCTGGAAAGATAC
	5′-AGCCCCTCCTCTTTCTTCCT
GAPDH	5′- GCCAAAAGGGTCATCATCTC
	5′- GGTGCTAAGCAGTTGGTGGT

Table2. Primers used for 3C-qPCR analysis of (A) P15 ^{INK4b}, (B) P16 ^{INK4a} and (C) and ERCC3

A: Primers used for 3C-q PCR along the INK4-ARF locus, fixed fragment at P15^{INK4b}

Constant primer	5′- CAGGTTGAGCAGGTTGGTTT
Probe	5′FAM- TCTAAAGCTTCACACTTGATCTTCCAAAGCCCCTT-3′BHQ^{R}
**EcoRI Fragment number**	**Forward primer Sequence**
1	5′- CCAACCTTAAACTACTGCTGAAAC
2	5′- GGAAGTCTGCCTATATGGGTTATC
3	5′- CTAAGGGGGTGGGGAGAC
4	5′- TGGTCATATTCAGTACTCACCTCA
5	5′- GTCCTGACTCCTACTCTGTTATCC
6	5′- CTGCCCAATACCTGTTCTCTTTC
7	5′- CCTTTCTTTCCTTAGGATGATGCC
8	5′- ATTATTCCTCCATTGCCTTTGCC
9	5′- GGAGACAGGACAGTATTTGAAGC
10	5′- GGGCAGCATTTGCTATCCTA

B: Primers used for 3C-q PCR along the INK4-ARF locus, fixed fragment at P16^{INK4a}

Constant primer	5′- CTGCCCCTTTGCTATTTTGC
Probe	5′FAM- CCGAACTTCTGCGGAGCTGTCGTC-3′BHQ^{R}
**EcoRI Fragment number**	**Forward primer Sequence**
1	5′- GGCTCCCATCAACATATCTAACTC
2	5′- CAGGTTGAGCAGGTTGGTTT
3	5′- CTAAGGGGGTGGGGAGAC
4	5′- GAGACGGGATTCCTCAACCAC
5	5′- GTCCTGACTCCTACTCTGTTATCC
6	5′- CTGCCCAATACCTGTTCTCTTTC
7	5′- CCTTTCTTTCCTTAGGATGATGCC
8	5′- ATTATTCCTCCATTGCCTTTGCC
9	5′- AGTGGAGCCTACAGTAATCATTTG
10	5′- TCTTTGGCTTCTATTCCCTAGA

C: Primers used for 3C-q PCR along the ERCC3 locus

Constant primer	5′- TCTTACCTGTTGGCCACTGACA
Probe	5′FAM- AGTTGTTCCTCCCAGGTCACATCCCAC-3′BHQ^{R}
Test primer	5′- GTCTGACCTTGCCCAGTGATAG
